# Peritumoral Microenvironment in High-Grade Gliomas: From FLAIRectomy to Microglia–Glioma Cross-Talk

**DOI:** 10.3390/brainsci11020200

**Published:** 2021-02-06

**Authors:** Roberto Altieri, Davide Barbagallo, Francesco Certo, Giuseppe Broggi, Marco Ragusa, Cinzia Di Pietro, Rosario Caltabiano, Gaetano Magro, Simone Peschillo, Michele Purrello, Giuseppe Barbagallo

**Affiliations:** 1 Department of Neurological Surgery, Policlinico “G. Rodolico-S. Marco” University Hospital, 95121 Catania, Italy; cicciocerto@yahoo.it (F.C.); simone.peschillo@gmail.com (S.P.); giuseppebarbagal@hotmail.com (G.B.); 2 Interdisciplinary Research Center on Brain Tumors Diagnosis and Treatment, University of Catania, 95123 Catania, Italy; dbarbaga@unict.it (D.B.); mragusa@unict.it (M.R.); purrello@unict.it (M.P.); 3 Department of Biomedical and Biotechnological Sciences—Section of Biology and Genetics Giovanni Sichel, University of Catania, 95123 Catania, Italy; dipietro@unict.it; 4 Department of Medical and Surgical Sciences and Advanced Technologies “G. F. Ingrassia”, Anatomic Pathology, University of Catania, 95123 Catania, Italy; giuseppe.broggi@gmail.com (G.B.); rosario.caltabiano@unict.it (R.C.); g.magro@unict.it (G.M.); 5 Oasi Research Institute—IRCCS, 94018 Troina, Italy

**Keywords:** glioma, glioblastoma, supratotal resection, 5-ALA, microglia, microRNA, extracellular vesicle, mTOR, immunomodulation

## Abstract

Cellular composition and molecular signatures of the glioma core compared with infiltrative margins are different, and it is well known that the tumor edge is enriched in microglia. In this review of the literature, we summarize the role of the peritumoral area in high-grade gliomas (HGGs) from surgical and biological points of view. There is evidence on the dual role of microglia in HGGs—a scavenger-tumoricidal role when microglia are activated in an M1 phenotype and a role favoring tumor growth and infiltration/migration when microglia are activated in an M2 phenotype. Microglia polarization is mediated by complex pathways involving cross-talk with glioma cells. In this scenario, extracellular vesicles and their miRNA cargo seem to play a central role. The switch to a specific phenotype correlates with prognosis and the pathological assessment of a specific microglial setting can predict a patient’s outcome. Some authors have designed an engineered microglial cell as a biologically active vehicle for the delivery of intraoperative near-infrared fluorescent dye with the aim of helping surgeons detect peritumoral infiltrated areas during resection. Furthermore, the pharmacological modulation of microglia-glioma cross-talk paves the way to more effective therapies.

## 1. Introduction

One of the most debated neurosurgical issues in the last few years has been the use of an aggressive resection beyond glioma margins [1,2,3,4]. A question concerning the usefulness of aggressive surgery was raised by the evidence that recurrences occur generally in the peritumoral areas [5]. Nevertheless, the present literature does not clearly define what is “supratotal resection” (SupTR), especially in high-grade gliomas (HGGs) [6]. Some authors have taken into consideration the FLAIR hyperintensity region beyond the enhancing nodule (EN), and it has been proven that this area has different biological features [6,7,8,9,10,11]. Ross et al. demonstrated that glioblastoma (GBM) has three principal tumoral microenvironments—the perinecrotic region, bulk tumor (corresponding to EN), and the infiltrative tumor margin (partially corresponding to FLAIR hyperintensity areas). Some authors say that 5-aminolevulinic acid (5-ALA) is a useful tool to differentiate GBM tumoral microenvironments and consequently their differential protein expression patterns [12]. The role of 5-ALA as an aid to reach the SupTR of GBMs [10,13] has already been reviewed. Nevertheless, to better understand if this field of research could have a valid biological substrate, it would be useful to study the biology of the tumor with special attention paid to the migration of tumoral cells and the cross-talking between glioma and microglia.

Nowadays, thanks to recent scientific acquisitions, it is well known that the cellular composition and molecular signatures of the glioma core compared with the infiltrative margins are really different and that the tumor edge is enriched in microglia. Yasargil supposed that glioma cells migrate faster in white matter [14], and there is now evidence on the role of microglia and their association with increased tumor migration and proliferation [15]. In this study, we summarize the role of peritumoral areas in HGG from surgical and biological points of view, highlighting the relationship between microglia and glioma cells. After a review of the literature, we show their biological features and explore future therapeutic possibilities.

## 2. Materials and Methods

On 17 November 2020, the PUBMED electronic database was searched, the following terms were applied: (microglia AND glioma AND microRNA OR supratotal resection OR FLAIRectomy). Results were analyzed with the PRISMA statement and processed with the ZOTERO reference manager (Center for History and New Media, George Mason University, Virginia, VA, USA). All papers written in languages other than English were excluded. Time or publication status restrictions were not applied. We selected the following:

- all clinical studies reporting the role of SupTR in HGGs excluding LGG; and
- all basic research concerning microglia-glioma cross-talk in peritumoral areas of HGGs focusing on the role of microRNAs.

## 3. Review

A total of 61 articles were identified using the search algorithm on PUBMED. Titles and abstracts of the 61 articles were reviewed, and 28 were excluded. The remaining 33 full texts were screened through the above-mentioned criteria. Of the 33 records identified, only 25 were selected, including 10 clinical articles and 15 original articles of basic research.

### 3.1. Histopathological Assessment of Microglia in the Central Nervous System (CNS) and Microglia–Glioma Cross-Talk in Peritumoral Areas

It has been largely demonstrated that the tumor microenvironment plays an active role in regulating tumor growth and progression [16]; in this regard, although the oncosuppressive function of cytotoxic tumor-infiltrating lymphocytes (TILs) has been well established in non-CNS tumors and their histopathological evaluation has now been fully included in pathologists’ practice [17], there is not the same evidence for the prognostic relevance of TILs in HGGs [18]. Moreover, the majority of glioma-infiltrating immune cells is not made up of lymphocytes but rather of microglia and macrophages to such an extent that HGGs are generally classified as lymphocyte-depleted neoplasms [19]. In recent years, although the mechanisms of recruitment and potential impact on patient survival of glioma-associated microglia and macrophages (GAMs) has been widely investigated, many aspects still remain to be explored in this field [20].

Microglial cells are a crucial part of the innate immune system within the brain and play a central role in the synaptic architecture, neurogenesis, and reaction after brain tissue damage [21]. At the end of complete brain development, microglia are confined by the blood–brain barrier (BBB) and become an autonomous cell population with self-renewal ability without any significant input from circulating blood cells. Microglia amount to about 5–20% of the overall glial cells resident in the healthy brain where they are ununiformly found in each region. More microglial cells are present in gray matter than white matter. A high concentration of microglia is present in the hippocampus, basal ganglia, the olfactory telencephalon, and the substantia nigra. Low concentration areas, instead, include fiber tracts, the cerebellum, and most of the brainstem. The cerebral cortex, thalamus, and hypothalamus have average cell densities. Microglia morphology is variable—in the white matter, microglial cells show elongated somata, and the processes are oriented along the fiber tracts; in the circumventricular organs, in contrast, they have a more compact morphology within the gray matter where microglia present many arbored and radially oriented processes [22].

**Figure 1:**
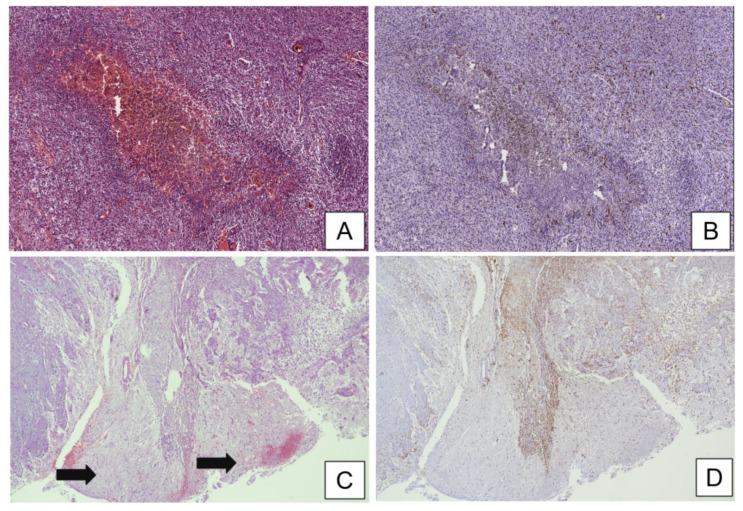
(**A**) Histological examination of glioblastoma (GBM) tissue sample showing a central focus of pseudopalisading necrosis, enriched with hemosiderin deposits (hematoxilin and eosin; original magnification 100×). (**B**) Immunohistochemical tests showing an abundant microglial activation, consisting of numerous clusters of differentiation (CD)163-positive glioma-associated microglia and macrophages (GAMs) that crowd the hypercellular zone surrounding the pseudopalisading necrosis (immunoperoxidase staining; original magnification 100×). (**C**) Histological detail showing fragments of unaffected brain parenchyma (arrows) at the periphery of a “classic-type” GBM, diffusely infiltrated by glioma cells (hematoxilin and eosin; original magnification 100×). (**D**) Immunohistochemical staining with CD163 highlights the presence of marked microglial activation with the M2-like phenotype at the invasive front of the tumor (immunoperoxidase staining; original magnification 100×).

On hematoxylin and eosin (H&E) stained sections, microglial cells have an elongated shape and dark and spindled nuclei; however, they are so scattered and small that they are very difficult to recognize in non-pathologic conditions. Microglia often present a similar morphology to that of the frequently found tangential or en face sections of endothelial cells, which similarly show elongated and dark nuclei. Ancillary methods, such as histochemistry (HC) and immunohistochemistry (IHC), allow better visualization of microglial cells because they highlight their dendritic processes [23]. In this regard, microglia is often visualizable by lectin histochemical staining and is typically positive for immunohistochemical markers of histiocytic lineage, including cluster of differentiation-68 (CD68), 163 (CD163), 206 (CD206), and ionized calcium-binding adaptor molecule 1 (Iba-1) [24] (Figure 1). While the presence of microglia is rarely detectable in a healthy brain, its amount and function become preponderant in response to parenchymal injury [23,25]. Two architectural variants of microglial activation are generally recognized—microglial nodules/stars and diffuse microgliosis; while the former appear as well-defined hypercellular nodules, composed both of astrocytes and elongated microglial cells (also called “rod cells”), and are typically associated with infectious diseases [26], the latter lack nodular structures and the rod-shaped microglial nuclei increase in number so much that they are easily identifiable and diffusely infiltrate brain tissue on H&E stained sections. Diffuse microgliosis may be histopathologically found in a variety of CNS diseases, including ischemia and tumors [27]. The evidence that the number of GAMs far exceed that of TILs has given rise to the suggestion that GAM–glioma cross-talk induces an immunosuppressive tumor microenvironment promoting glioma growth. It has been found that IL-33, because of its strong correlation with increased GAMs, plays a proinflammatory role in the tumor microenvironment and thus promotes tumorigenesis in HGGs. Moreover, decreased IL-33 expression has been associated with better overall survival and tumor growth inhibition [28].

Characterization of the cellular population composition of the HGG core versus infiltrative margins reveals that the peritumoral areas are enriched in microglia, and it has been associated with increased tumor migration [15,29,30]. Microglia are the largest population of peritumoral areas, contributing to the total tumor mass by at least one third [31]. In 1925, Wilder Penfield hypothesized that there is a strict link between microglia and glioma cells. He studied the development and behavior of microglia and published a paper in which he suggested that microglia play an important role in extracellular matrix (ECM) remodeling [32]. Nowadays, it is clear that microglia are recruited by tumoral cells thanks to the secretion of different factors such as chemokines, cytokines, etc. One of the principal chemokines involved is chemokine C–C motif ligand 2 (CCL2) that recruits microglial cells through CCR2 and plays a crucial role in promoting tumor growth, neo-angiogenesis and invasiveness, stimulating microglial cells to produce IL-6 [33]. There is evidence concerning the dual role of microglia in HGG—a scavenger-tumoricidal role when microglia are activated in an M1 phenotype and, on the contrary, a role favoring tumor growth and infiltration/migration when microglia are activated in an M2 phenotype. Lisi et al. demonstrated that in the presence of a tumor microenvironment, microglia shift into the activated M2 phenotype, which is associated with neuroprotection and tumor growth stimulation [21]. Microglial cells undergo a different pattern of activation depending on the glioma stage of the disease; in the early stage of pathology, microglia are exposed to basal glioma-derived factors, increasing their M2 polarization status. In the late stage of pathology, in contrast, the presence of inflammatory activated glioma-derived factors stimulates the polarization into M1 phenotypes [34].

Juliano et al. confirmed that glioma cells induced microglial activation and that microglia speed was correlated strongly with the local density of glioma cells. Therefore, glioma cells stimulate the motility of microglial cells at the peritumoral infiltrative margins but these two cellular populations showed very different migratory behavior, even when moving through the same microenvironment. It is unclear if glioma cells and microglia are either responding to different migratory cues or are responding to the same cues but in different ways. Microglia and glioma migration pathways are different. Microglia move in a random way, whereas glioma cells exhibit a “committed” migratory behavior with significantly increased directionality compared with microglia. After activation, microglia may enable more contact with cells with this random migration in a short period of time, resembling a surveillance function [15].

### 3.2. MicroRNAs (miRNAs) Are Extensively Dysregulated in GBM

miRNAs are at the forefront of current biomedical research because they are master regulators of gene expression within cells (both in physiological and pathological conditions), allow intercellular communication, and are promising diagnostic, prognostic, and therapeutic biomarkers [35,36,37,38]. The first report regarding the extensive dysregulation of miRNA expression in GBM was from Ciafrè et al. By investigating the global expression of 245 miRNAs from nine primary GBM patients, these authors identified nine (miR-10b; miR-130a; miR-221; miR-125b-1; miR-125b-2; miR-9-2; miR-21; miR-25; and miR-123) upregulated and four (miR-128a; miR-181c; miR-181a; and miR-181b) downregulated miRNAs compared with normal brain parenchyma [39]. Since then, some of these miRNAs have been confirmed as dysregulated and characterized as functionally involved in the control of critical biological functions (from apoptosis (e.g., miR-21) [40] to cell cycle (e.g., the cluster miR-221/222, residing within the X chromosome) [41,42] in glioma cells. Other studies extended the parterre of dysregulated miRNAs in GBM, identifying new candidates to be studied for their functional involvement in this cancer. Silber et al. identified miR-124 and miR-137 as downregulated in glioma stem cells and involved in the maintenance of their stemness [43], and Kefas et al. defined miR-7 as a tumor suppressor in GBM, regulating cell viability and invasiveness of cancer cells by targeting the Epidermal Growth Factor Receptor (EGFR) [44]. The first evidence that miRNAs can be found also in extracellular body fluids, incorporated into microvesicles, or bound to specific RNA-binding proteins, came from Skog et al. [45]. This study paved the way for the use of miRNAs as potential non-invasive diagnostic biomarkers for GBM [46,47,48,49,50,51]. The involvement of miRNAs in GBM cell resistance to chemotherapy was first reported by Li et al. [52]; they showed that oncomiR-21 is involved in GBM cell resistance to the chemotherapeutic agent teniposide by targeting LRR binding FLII interacting protein 1 (LRRFIP1) mRNA. Later, Ujifuku et al. reported miR-195, miR-455-3p, and miR-10a* as involved in the resistance of GBM cell line U251 to temozolomide [53]. miRNA expression profiles have also been used to classify GBM into clinically and genetically distinct subtypes, matched to specific neural precursor cell types, as reported by Kim et al. [54]. The prognostic significance of specific miRNA signatures or polymorphisms has also been described [55,56,57,58,59,60]. A more comprehensive summary of miRNAs involved in GBM pathogenesis is shown in Table S1.

### 3.3. miRNAs Show Specific Patterns of Expression in GBM Core and in the Peritumoral Area

Notwithstanding the extensive characterization of the transcriptome and proteome of the peritumoral area [61,62,63,64,65,66], little is known about the expression and involvement of miRNAs in this area and, more specifically, their role in the cross-talk between GBM and microglial cells. One of the first studies on the involvement of miRNAs in the pathogenesis of GBM was conducted by comparing their expression between the central tumor area, surgically and histopathologically recognized as frankly tumoral, and the peripheral glial area, without any evidence of tumor presence, by a surgeon’s macroscopical evaluation [39]. In the same study, an intermediate region located between frankly tumoral and peripheral glial areas had also been assayed. Since that publication, it has been clear that the peritumoral area has a proper distribution of miRNAs that only partially resembles that of the bulk tumor, with miRNAs miR-10b, miR-130a, miR-221, miR-125b-1, miR-125b-2, miR9-2, miR-21, miR-25, and miR-123 upregulated, and miR-128a and three members of the miR-181 family (miR-181a/b/c) downregulated in the central tumor area compared with the peripheral glial area. Later, Godlewski et al. found another set of miRNAs differentially expressed (DE) between the central tumor area compared with the adjacent tumor area [67]. In this case, no indication about the precise location of the adjacent tumor area was indicated by the authors. Two miRNAs (miR-21 and miR-10b) were confirmed as upregulated in the central tumor area compared with the peripheral region also in this study—this is another indication of how tumor heterogeneity and sampling may affect downstream miRNA expression analysis, notwithstanding the fact that some miRNAs confirm their critical role as oncomiRNAs (e.g., miR-10b expression correlated with multifocal lesions on enhanced MRI, confirming its involvement in the invasion capability of GBM cells, as described by Sasayama et al.) [68]. Fazi et al. found a plethora of miRNAs DE among white matter, bulk tumor, and peritumoral areas [69]; some of them were upregulated in the frankly tumoral mass versus peritumoral area (miR-21-3p, miR-196b-5p, miR-135b-5p, and miR-183-3p known as “oncomiRs” in several tumors, including GBM), and others were downregulated in the same comparison (miR-219a, miR-338-3p, and miR-338-5p, with an established role in oligodendrocyte maturation, and miR-34b and miR-34c, widely recognized as tumor suppressor miRNAs in general and specifically in GBM). Some miRNAs were commonly dysregulated in a frankly tumoral mass and peritumoral area versus the healthy white matter (e.g., upregulated “oncomiRNAs” miR-106b and miR-93). Differential patterns of miRNA expression were also observed by the authors between the infiltrated peritumor area and the non-infiltrated peritumor area (e.g., miR-182-5p, miR-183-5p, and miR-96-5p). In another study, Piwecka et al. found that miR-625, a known tumor suppressor involved in the invasion and migration of gastric cancer cells [70,71], was down expressed in the comparison between peritumoral area and healthy white matter, but it did not show any differential expression between the bulk tumor and healthy tissue [72]. Hide et al. identified a signature made of five (miR-219-5p, miR-219-2-3p, miR-338-3p, miR-27b, and miR-23b) and seven (miR-630, miR-1246, miR-642b, miR-1181, miR-H18, miR-3195, and miR-3663-3p) miRNAs up- and downregulated, respectively, in the peritumoral area as compared with the frankly tumoral area [73]. The same authors focused on miR-219-5p whose expression in the border of the tumor was linked to the presence of oligodendrocyte lineage cells. Furthermore, by using oligodendrocyte precursor cell (OPC) or GBM cell-conditioned media, the authors demonstrated how tumor cells can stimulate OPC growth while the latter may induce the expression of stemness and chemoradioresitance-related genes within tumor cells, leading toward a pro-oncogenic microenvironment at the border of GBM, called the “border niche.” For all these reasons, miRNAs appear to play a master role in the progression of GBM and a comprehensive view of their involvement in the cross-talk between GBM and tumor microenvironment appears equally important to better explain the pathogenesis of this cancer. A summary of DE miRNAs whose expression had been studied in the tumor core and in the peritumoral area is reported in Table 1.

### 3.4. miRNAs Mediate the Cross-Talk between GBM and Microglia Cells

**Figure 2:**
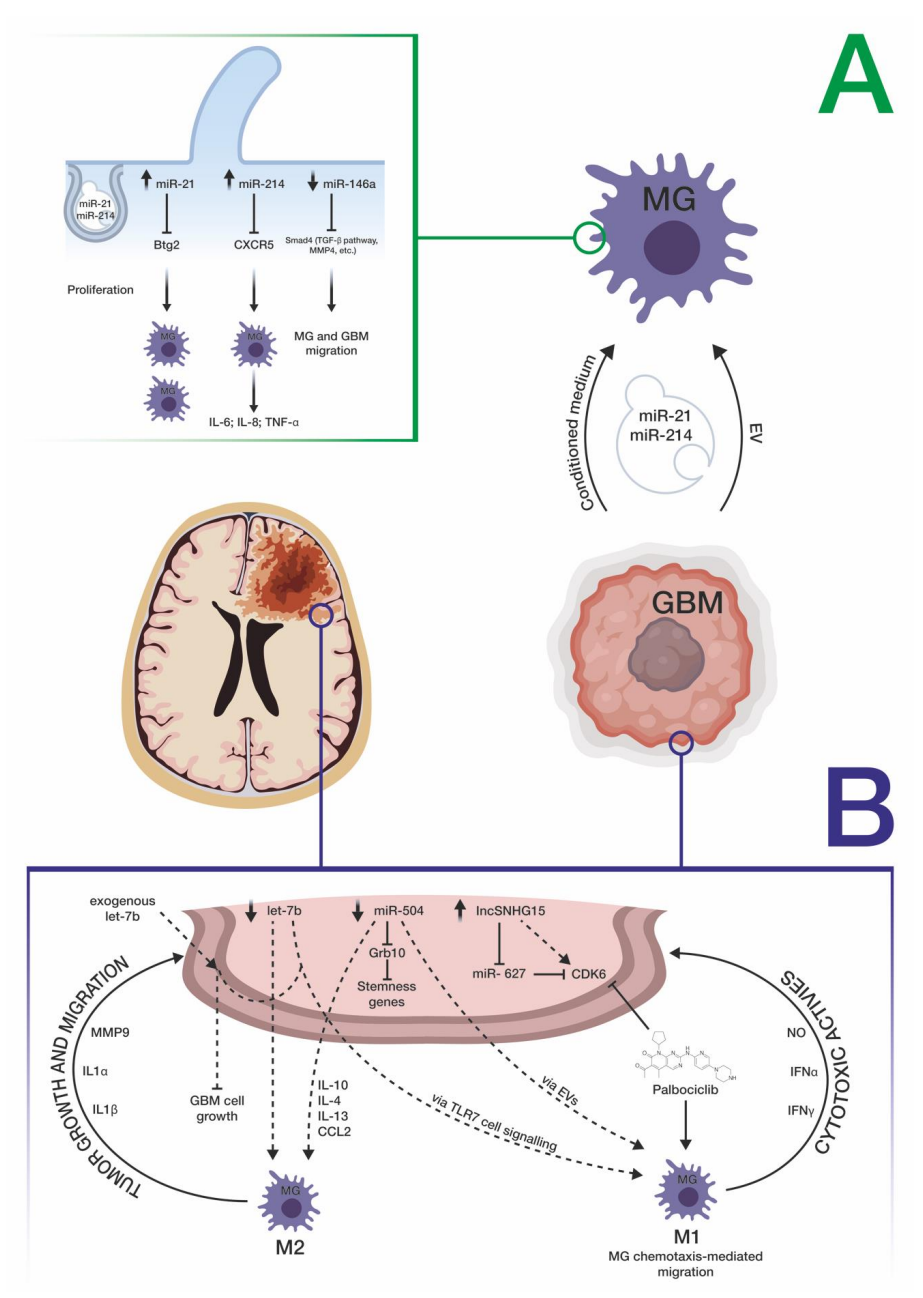
Schematic representation of miRNA-mediated cross-talk between GBM and microglia cells. (**A**) Effects of the cross-talk between GBM and microglia in pathological conditions, inducing microglial M2 polarization. (**B**) Suggested miRNA-mediated therapeutic strategies inducing the switch from M2 to M1 microglial polarization. Refer to the text for a more detailed description. MG = microglia; GBM = glioblastoma.

One of the first pieces of evidence of the cross-talk between GBM and microglial cells comes from the study led by Van der Vos et al. on the uptake of GBM-derived extracellular vesicles (EVs) by microglial cells [74,75]. Through combined in vitro and in vivo approaches, these authors demonstrated that the uptake of GBM-derived EVs by microglial cells led to the internalization of miR-21 and miR-451, two known oncogenic miRNAs enriched within GBM EVs, into the latter cell types. This uptake led to decreased levels of the mRNA of the pleiotropic gene c-Myc, a target common to both miRNAs, demonstrating downstream functional effects of the internalization of GBM miRNAs into microglial cells. The same authors speculated that this internalization could also lead to a switch of microglial cells versus a tumor-supportive phenotype through the secretion of immunosuppressive cytokines. Abels et al. supported the critical role played by GBM EV-mediated transport of miR-21 within microglial cells [76]—in an in vivo mouse model, this transfer exerted the downregulation of the mRNA transcribed by the BTG anti-proliferation factor 2 (Btg2) gene, thus stimulating microglial cell proliferation. The GBM microenvironment, reshaped through these modifications, may contribute to tumor progression. Other evidence on the cross-talk between GBM and microglia comes from the finding reported by Yang et al.; they demonstrated that miR-214-5p, aberrantly upregulated in GBM cells, can be transferred to microglia, through exosomes, contributing to the suppression of microglial C–X–C motif chemokine receptor 5 (CXCR5), and, consequently, increasing the microglial secretion of inflammatory cytokines IL-6, IL-8, and TNF-α, which, in turn, favor a tumor-supportive microenvironment [77]. The microglial function appears to be modulated also by miRNAs belonging to the let-7 family [78]. In detail, Bonfiglioli et al. found that a specific sequence motif (*UUGU*), characterizing some of the let-7 family members, determined the activation of an M1-like microglial phenotype, through the interaction with Toll-like receptor 7 (TLR7), which can trigger an anti-tumoral microenvironment at the periphery of the GBM cell mass: this is in agreement with the downregulation of let-7 miRNAs observed in GBM cells and with the correlation between their down expression and a poor prognosis in both human and murine GBM [79,80]. Karthikeyan et al. demonstrated how microglial cells exposed to GBM conditioned-medium exhibited a greater ability to migrate and attributed this phenotype to decreased levels of miR-146a and resulting upregulation of its target SMAD family member 4 (SMAD4), a critical node involved in the activation of the TGF-β pathway and genes such as matrix metallopeptidase 9 (MMP9), which facilitates tumor cell invasion [81]. A reverse cross-talk, between miR-124-3p-containing microglial exosomes and neuronal cells, was demonstrated by Li et al. in a mouse model of brain injury [82]; this brain-specific miRNA is downregulated both in activated microglia and in GBM cells [43,83,84]. Li et al. demonstrated that temozolomide (TMZ)-resistant GBM cells trigger the M2-polarization of microglial cells thanks to the long-noncoding RNA SNHG15 (upregulated in GBM cells), and its associated molecular axis made up of miR-627 (tumor suppressor, normally downregulated in GBM) and CDK6 (oncoprotein, directly targeted by miR-627) [85]. The same authors suggested the use of Palbociclib, a CDK6 inhibitor, to overcome TMZ resistance and to shift microglial cells towards an M1 polarization. Another way of miRNA-mediated cross-talk between GBM and microglial cells was elucidated by Bier et al., who demonstrated how miR-504, normally downregulated in both GBM and glioma stem cells compared with healthy white matter, may be transferred to microglial cells, allowing their M1 polarization, thanks to EV cargo [86] (Figure 2). A summary of miRNAs involved in the cross-talk between GBM cells and microglia is reported in Table 2.

### 3.5. Role of Surgery in Peritumoral Infiltrated Areas

While it is well established that SupTR of the FLAIR hyperintensity zone on MRI guarantees a better prognosis in patients affected by LGG, the safety and efficacy of an aggressive tumor removal beyond EN margins remain a matter of debate for HGGs [1,3]. The major concerns of many neurosurgeons are about the inconvenient onco-functional balance because of the high risks of postoperative neurological complications. Some authors demonstrated the superiority of lobectomy compared with tumorectomy in non-eloquent HGGs in two recent retrospective series. They demonstrated that, in patients with completely resectable, non-eloquent area GBMs, SupTR provides better survival without a negative impact on neurological performance [87,88]. For the first time, Li et al. focused attention on the peritumoral infiltrated FLAIR hyperintensity areas and analyzed retrospectively a series of 1229 patients affected by GBM. Their experience showed that an extent of resection (EOR) > 53% of the FLAIR hyperintensity beyond the EN was associated with longer survival compared with controls (patients who receive an EOR <53% of FLAIR areas) [2]. One year later, in a retrospective series in which 282 patients treated for GBM were analyzed, Pessina et al. found the same result with a different FLAIRectomy threshold conditioning survival (45% and not 53%) [4]. Other study groups, instead, in a series of 245 and 64 patients did not find a survival improvement with FLAIRectomy [3,7]. On the contrary, we recently described in our single-center experience on 68 patients that a FLAIR-based EOR, in multivariate analyses comprising age, isocitrate dehydrogenase 1 (IDH-1) mutation, O6 methylguanine methyltrasferase (MGMT)-methylation, Radiotherapy (RT) dose, and the number of temozolomide cycles, appears to be a stronger survival predictor compared with EN resection [9]. In a detailed analysis of 585 cases, Jang et al. found that HGGs probably explain the literature discrepancies. The authors evaluated the FLAIR hyperintensity regions and clarified that HGGs should be divided into two main classes based on the Volume_FLAIR_/Volume_EN_ ratio. Patients with Volume_FLAIR_/Volume_EN_ < 10 are defined “proliferation-dominant” subtype, while HGGs with Volume_FLAIR_/Volume_EN_ > 10 are defined “diffusion-dominant” subtypes. The authors showed a prognosis improvement associated with FLAIR resection beyond the EN in “proliferation-dominant” IDH-1 mutated HGGs, while they did not find a correlation between SupTR of EN and survival in “diffusion-dominant” IDH-1 wild type HGGs [89]. Moreover, Stummer proposed to shift the surgical target from “conventional” neuroimaging to “metabolic” imaging using 18 F-fluor-ethyl-tyrosine-PET (18 F-FET-PET) to identify the peritumoral areas of surgical interest. He described that postoperative 18F-FET-PET volumes beyond MRI EN predict Overall Survival (OS) and Progression Free Survival (PFS) in patients surgically treated for GBMs. He furthermore stated that 5-ALA guided resection beyond EN leads to less postoperative 18F-FET-PET tumor [13], improving survival. Regarding the effects of SupraTR of HGGs in neurological and neurocognitive fields, Sarubbo et al. proposed that awake surgery can improve survival preserving the quality of life [90].

### 3.6. Where We Are Going

The study of microglia could open the way for effective diagnostic, prognostic, and therapeutic approaches. From a diagnostic and prognostic point of view, for example, Zeiner et al. evaluated the differential immunoexpression of selected microglial markers on a series of 344 WHO grade I-IV gliomas and further validated their findings on a cohort of 241 IDH-wildtype WHO grade IV GBMs, correlating the differential GAM expression to patient prognosis. In this regard, the following immunomarkers were studied: Iba1 (pan-GAM marker), CD68 (pan-GAM markers), CD163 (M2 phenotype GAM marker), and CD206 (M2 phenotype GAM markers). They found that IDH-wildtype GBMs contained mixed M1-M2 phenotype GAMs and higher levels of CD68+, CD206+, and CD163+ GAMs infiltrating non-necrotic tumor areas were associated with better prognosis [24]. It has also been shown that GBM areas containing pseudo-palisading necrosis (PPN)—a histopathological hallmark of HGGs—were particularly crowded with GAMs that had migrated to necrotic foci to phagocytose cell debris. GAMs populating the PPN were elongated in morphology at the hypercellular area of the necrotic area and showed CD163 expression, suggesting a switch to the M2-phenotype [91].

**Figure 3:**
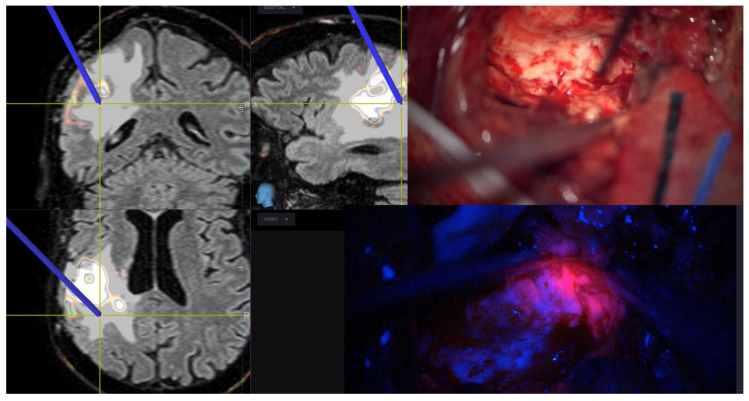
Images show an intraoperative view of peritumoral areas. In the neuronavigational view, the tracer is placed in the FLAIR hyperintensity zone beyond the enhancing nodule (EN). In the right upper image, there is the corresponding and apparently normal tissue under white light and in the image at the bottom the same surgical site under blue light revealing the presence of lava-like fluorescence.

It is well known that fluorescence-guided surgery for HGG is an effective intraoperative tool that can provide real-time information distinguishing tumoral tissue from normal brain tissue. There are three most commonly used compounds—5-ALA, sodium fluorescein, and indocyanine green video-angiography. Among them, 5-ALA is the only metabolic tracer and it is consequently the only drug able to detect directly tumoral cells. It is a non-fluorescent prodrug, the precursor of the heme synthesis pathway and it is first absorbed by tumoral cells and then converted into a fluorescent protoporphyrin IX (PpIX). When placed under blue-violet light, PpIX is able to return red light in the visible spectrum frequencies. In the last few years, the role of 5-ALA in detecting infiltrating peritumoral areas beyond EN has been studied [92,93]. A recent phase II clinical trial correlated cellularity with fluorescence intensity in HGG. With this study, the authors demonstrated a strict correlation between the intensity of 5-ALA and the number of proliferating tumoral cells [94]. Various published studies confirm the direct correlation between the use of 5-ALA and the achievement of a SupTR of EN [10,13,95] (Figure 3). However, different authors also highlighted the possibility of false positives, particularly in peritumoral areas [94,96]. Guo et al. produced engineered microglial cells, BV2, as biologically active vehicles for delivery of intraoperative near-infrared fluorescent dye DiD (DiDBV2-Fe). To assess the fluorescence-guiding potential of DiDBV2-Fe, the authors tested its biological properties in vitro (U87MG cells) and in vivo using an orthotopic GBM model. They demonstrated in a laboratory setting that treatment with DiDBV2-Fe produced a strong and selective tumor tropism in response to CCL2 secreted by U87MG tumor cells. The drug efficiently crossed the BBB, resulting in more than 90% fluorescence intensity generated by DiDBV2-Fe microglial cells being detected in the brain. Moreover, DiDBV2-Fe produced a clear tumor boundary delineation on near-infrared imaging exhibiting a superior tumor-to-brain fluorescence ratio to 5-ALA. Moreover, DiDBV2-Fe did not show any significant adverse effects in mice opening the way to search for a new safe and effective drug for intraoperative highlighting of tumor borders [97].

The tuberous sclerosis complex (TSC)-mTOR pathway regulates macrophage polarization. It seems that mTOR activation causes the polarization of microglia to the M2 subtype. The cross-talk between mTORC1 and mTORC2, occurring in microglia, guarantees a correct balance between cellular growth and division. The activation of mTORC1 generally increases the cellular capacity of protein and lipid biosynthesis, and inhibits macroautophagy, thus promoting anabolic processes. From a pharmacologic point of view, in preclinical murine models, minocycline (microglia suppressor) was demonstrated to be effective in the suppression of tumor growth and progression. Minocycline may block MMP expression, interfering with the remodeling of the extracellular matrix by microglia. Unfortunately, minocycline was not so effective when used in human clinical trials [98,99]. THIK-1, a K+ channel present on the microglia surface, has recently been described as a regulator of microglial motility, surveillance, and IL-1β release (well known to be involved in glioma angiogenesis and invasion). The THIK-1 channel has therefore been suggested as a target for glioma treatment but there is a lack of drugs targeting it today and there is a need for a better understanding of this pathway [100]. mTORC1 activities are deregulated in HGGs because of mutations in several oncogenes (PI3K, AKT, or Ras) and/or tumor suppressors (PTEN, LKB1, or TSC1/2), involved in mTORC1 control activation. In this scenario, Lisi et al. demonstrated in both early- and late-stage in vitro models that mTOR inhibition by RAPA and RAD prevents microglial polarization to the M2 subtype. Inhibition of mTOR in microglial cells leads to relevant antitumor effects mediated directly by the polarization of microglia to the M1 subtype with a cytotoxic effect and prevents proliferation avoiding the polarization to the M2 status. Microglial cells in the M1 status release cytokines, prostaglandins, and reactive oxygen intermediates, including nitric oxide [101]. These substances can have cytotoxic effects on tumoral cells. Nitric oxide, for example, plays a hyper-sensitization role in traditional chemo- and radiotherapy [102].

Finally, as GAMs are the major cellular component of the glioma microenvironment, their modulation plays a key role in influencing also the remaining cell components, including TILs. The antigen presentation function of GAMs needs to be further investigated and therapeutically targeted in order to offer new potential therapeutic options (vaccination studies and/or T cell checkpoint inhibitor drugs) [103]. Prospectively, the use of nanosensors and nanocarriers for the detection of GBM miRNAs delivered into the peritumoral area and for their transport into microglial cells with therapeutic purposes, respectively, may represent a new tool for the treatment of this disease [104,105,106].

## 4. Conclusions

The crucial battlefields of every war are the peripheral zones. The correct and extensive understanding of microglia–glioma cross-talk could help in understanding the physiopathology of this mysterious and complex disease, opening an important scenario for its treatment.

## Figures and Tables

**Table 1:** Summary of miRNAs differentially expressed (DE) between GBM core and peritumoral area.

DE miRNA (GBM Core vs. Peritumoral Area)	Expression (GBM Core vs. Peritumoral Area)	Year of Publication	Technique Used to Assay miRNA Expression	Reference (PMID)
miR-10b; miR-130a; miR-221; miR-125b-1; miR-125b-2; miR9-2; miR-21; miR-25; miR-123	Upregulated	2005	Microarray	16039986
miR-128a; miR-181c; miR-181a; miR-181b	Downregulated	2005	Microarray	16039986
miR-21; miR-10b	Upregulated	2008; 2009	Microarray	16039986; 19536818
miR-21-3p; miR-196b-5p; miR-135b-5p; miR-183-3p	Upregulated	2015	SAGE sequencing and qRT-PCR	26188123
miR-219a; miR-338-3p; miR-338-5p; miR-34b; miR-34c	Downregulated	2015	SAGE sequencing and qRT-PCR	26188123
miR-625	Downregulated in peritumoral area vs healthy white matter	2015	Microarray; small RNA deep sequencing	25864039
miR-219-5p; miR-219-2-3p; miR-338-3p; miR-27b; miR-23b	Downregulated	2018	Microarray	29559295
miR-630; miR-1246; miR-642b; miR-1181; hsv-miR-H18; miR-3195; miR-3663-3p	Upregulated	2018	Microarray	29559295

**Table 2:** Summary of miRNAs involved in the cross-talk between GBM and microglial cells.

miRNA Involved in the Cross-Talk between GBM Cells and Microglia	Functional Involvement of miRNA in the Cross-Talk	Year of Publication	Validated Target (Official Gene Symbol)	Technique Used to Assay miRNA Expression	Reference (PMID)
miR-21 and miR-451	Internalization of miR-21 and miR-451, two known oncogenic miRNAs enriched within GBM extracellular vesicles (EVs), into microglial cells. The consequence is a switch of microglial cells versus a tumor-supportive phenotype through the secretion of immunosuppressive cytokines	2016	MYC	qRT-PCR	26433199
miR-21	GBM EV-mediated transport of miR-21 exerted the downregulation of the mRNA transcribed by the BTG anti-proliferation factor 2 (Btg2) gene, thus stimulating microglial cell proliferation	2019	BTG	qRT-PCR; ddPCR	31533034
miR-214-5p	MiR-214-5p, aberrantly upregulated in GBM cells, can be transferred to microglia, through exosomes, contributing to the suppression of microglial C-X-C motif chemokine receptor 5 (CXCR5), and, consequently, increasing the microglial secretion of inflammatory cytokines IL-6, IL-8 and TNF-α	2019	CXCR5	qRT-PCR	30394221
let-7 UUGU motif	Let-7 family members containing UUGU motif determined the activation of an M1-like microglial phenotype, through the interaction with the Toll-like receptor 7 (TLR7), which can trigger an anti-tumoral microenvironment at the periphery of the GBM cell mass	2019	Physical interaction and activation of TLR7 in microglial cells	qRT-PCR	31825829
miR-146a	Microglial cells exposed to GBM conditioned-medium exhibited a greater ability to migrate. This was linked to downregulation of miR-146a and upregulation of its target SMAD family member 4 (SMAD4)	2018	SMAD4	qRT-PCR	29861845
miR-124-3p	MiR-124-3p is a brain-specific miRNA, downregulated both in activated microglia and in GBM cells. MiR-124-3p was demonstrated to contribute to communication between microglial and neuronal cells via microglial exosomes	2019	N/A	qRT-PCR	31190315
miR-627	Temozolomide-resistant GBM cells trigger the M2-polarization of microglial cells thanks to the long-noncoding RNA SNHG15 (upregulated in GBM cells) and its associated molecular axis made up of miR-627 (tumor suppressor, normally downregulated in GBM) and CDK6 (oncoprotein, directly targeted by miR-627)	2019	CDK6	qRT-PCR	31462285
miR-504	MiR-504, normally downregulated in both GBM and glioma stem cells, compared with healthy white matter, may be transferred to microglial cells, allowing their M1 polarization, thanks to EV cargo	2020	N/A	Microarray; qRT-PCR	33093452

## Supplementary Materials

The following are available online at https://www.mdpi.com/2076-3425/11/2/200/s1, Table S1: Summary of miRNAs involved in GBM.

## Abbreviations

HGGs	High-Grade Gliomas
SupTR	Supratotal Resection
EN	Enhancing Nodule
GBM	Glioblastoma
5-ALA	5-Aminolevulinic Acid
CNS	Central Nervous System
TILs	Tumor-Infiltrating Lymphocytes
GAMs	Glioma-Associated Microglia and Macrophages
BBB	Blood–Brain Barrier
H&E	Hematoxylin and Eosin
HC	Histochemistry
IHC	Immunohistochemistry
ECM	Extracellular Matrix
CCL2	Chemokine C–C Motif Ligand 2
miRNAs	MicroRNAs
EGFR	Epidermal Growth Factor Receptor
LRRFIP1	LRR Binding FLII Interacting Protein 1
DE	Differentially Expressed
OPC	Oligodendrocyte Precursor Cell
Evs	Extracellular Vesicles
Btg2	BTG Anti-Proliferation Factor 2
CXCR5	C–X–C Motif Chemokine Receptor 5
TLR7	Toll-Like Receptor 7
SMAD4	SMAD Family Member 4
MMP9	Matrix Metallopeptidase 9
TMZ	Temozolomide
IDH-1	Isocitrate Dehydrogenase 1
RT	Radiotherapy
OS	Overall Survival
PFS	Progression Free Survival
TSC-mTOR	Tuberous Sclerosis Complex
