# Supplementary material for: Peritumoral Microenvironment in High-Grade Gliomas: From FLAIRectomy to Microglia–Glioma Cross-Talk
Source: Brain Sci. 2021 Feb 6;11(2):200. doi: 10.3390/brainsci11020200 (PMC7915863; doi:10.3390/brainsci11020200)
Supplement: Supplementary file 1 [file brainsci-11-00200-s001.pdf]

Table S1. Summary of miRNAs involved in GBM. The column headed "expression" refers to up- or down-regulation of miRNAs in GBM tissues or cell lines compared with healthy brain parenchyma or control cell lines; any particular type of comparison is otherwise specified in the table. The references referring to the main topic of the review (the crosstalk between GBM multiforme and microglia cells) are in bold. MV = microvesicle; N/A = not annotated

miRNA	Expression (GBM cells vs unaffected brain)	Year of publication	Functional role of miRNA (if characterized)	Validated target (official gene symbol)	Technique used to assay miRNA expression	Study Reference (PMID)
miR-10b; miR-130a; miR-221; miR-125b-1; miR-125b-2; miR9-2; miR-21; miR-25; miR-123	Upregulated	2005	N/A	N/A	Microarray	**16039986**
miR-128a; miR-181c; miR-181a; miR-181b	Downregulated	2005	N/A	N/A	Microarray	**16039986**
miR-21	Upregulated	2005; 2008; 2009; 2011; 2012; 2013; 2014; 2017; 2019; 2020	Antiapoptotic; positive control of cell migration and invasion; resistance to chemotherapy	RECK; TIMP3; PDCD4; LRRFIP1; PTEN; STAT3; IGFBP3	Microarray; qRT-PCR; northern blotting	16024602; 18591254; 19013014; 22964638; 19559015; 21636706; 28534371; **31533034** 33123586; 22709411; 25059666
cluster miR-221/222	Upregulated	2007; 2008; 2009; 2010 ; 2012; 2013	Positive regulators of cell cycle and cell migration	CDKN1B;CDKN1C; NAIP; BBC3; PTPRM; GJA1; MGMT	Microarray; qRT-PCR; northern blotting	17721077; 18759060; 18413744; 20813046; 22294051; 24147153

miR-124 and miR-137	Downregulated	2008; 2011; 2012; 2013; 2015; 2016	Maintainance of glioma cell stemness; negative regulator of cell migration; proliferation and invasion	CDK6; PTGS2; PPP1R13L; GLIPR1; SOS1; RAC1; EZH2; AURKA	Highthorughput and lowthorughput qRT-PCR	18577219; 21196113; 22406049; 23624869; 23714687; 23817964; 25310349; 25939439; 28242198
miR-7	Downregulated	2008; 2009	Negative regulator of GBM cell viability and invasiveness	EGFR; RAF1	qRT-PCR	18483236
let-7a; miR-15b; miR-16; miR-19b; miR-21; miR-26a; miR-27a; miR-92; miR-93; miR-320; miR-20	Detected in serum MVs from GBM patients	2008	N/A	N/A	qRT-PCR	19011622
miR-128	Downregulated	2008; 2010; 2011; 2013	Negative regulator of glioma self-renewal	BMI1; ANGPTL6; PDGFRα; EGFR; SUZ12	Microarray; qRT-PCR	**19010882**; 19941032; 21874051; 23733246
miR-451	Downregulated; Upregulated in GBM non-stem cells vs GBM stem cells	2008; 2010	Negative regulator of cell growth and viability (synergy with Imatinib to block GBM cell growth and viability)	N/A	Microarray	18765229; 20816946
miR-34a	Downregulated	2009; 2012; 2013	Negative regulator of cell proliferation; cell cycle progression; cell survival; and cell invasion	MET; NOTCH; EGFR; PDGFRA	qRT-PCR	19773441; 22580610; 22750848

miR-10b	Upregulated	2009; 2011; 2012	Associated with multifocal lesions; positive control of tumor cell growth	HOXD10; BCL2L11; TFAP2C; CDKN1A; CDKN2A; TP53; FOXO3; CYLD; PAX6; PTCH1	qRT-PCR	**19536818**; 21471404; 23034333
miR-196a	Upregulated	2010	N/A	N/A	Microarray	20601442
let-7	Downregulated	2011	Negative regulator of cell proliferation and migration	KRAS	N/A	20607356
miR-195; miR-455-3p and miR-10a*	Upregulated in themozolomide resistant vs sensitive GBM cells	2010	Resistance to themozolomide	N/A	Microarray	20444541
miR-93	N/A	2011	Positive control of angiogenesis	ITGB8	qRT-PCR	20956944
miR-146b-5p	Downregulated	2010; 2013	Negative control of cell migration and invasion	EGFR; MMP16	qRT-PCR	20874002; 23796692
miR-145	Downregulated both in GBM vs normal brain and in GBM stem cells vs GBM non-stem cells	2012; 2013; 2014	Positive control of cell differentiation	OCT4; SOX2; NEDD9; CTGF; ABCG2	Microarray	22098779; 22869051; 23390502
miR-205	Downregulated	2011	Negative regulator of apoptosis; cell cycle; cell viability; cell invasion	VEGFA	qRT-PCR	22159356
miR-9/9*; miR-17	Highly expressed in GBM stem cells	2011	Positive regulators of cell stemness	CAMTA1	Microarray; qRT-PCR	21857646
miR-155	Upregulated	2012; 2013	Positive regulator of cell growth	GABRA1; MXI1	Microarray; qRT-PCR	22470130; 24376632

miR-125b	Upregulated; Downregulated in GBM stem cells vs GBM non-stem cells	2009; 2010; 2012; 2013; 2014; 2015; 2016; 2019	Positive regulator of cell proliferation and growth; inhibitor of apoptosis; resistance to themozolomide and TNF-related apoptosis-inducing ligand (TRAIL)	BMF; CDK6; CDC25A; E2F2; MAZ; GJA1; TNFAIP3; NKIRAS2; PIAS3; BAK1; MAPK14	qRT-PCR	19471102; 19948152; 21879257; 22999819; 22415301; 24046143; 23835866; 24901050; 23857508; 24356103; 24643683; 26170223; 27698350; 31056533; 24169356
miR-31	Downregulated	2012	Inhibition of cell migration and invasion	RDX	qRT-PCR	22089331
miR-483-5p	Downregulated	2012	Negative regulator of cell proliferation	MAPK3	Microarray	22465663
miR-218	Downregulated	2012	Negative regulator of cell invasion	LEF1	qRT-PCR	22766851
miR-196b	Upregulated	2012	Positive regulator of cell proliferation	N/A	Microarray	22723849
miR-183	Upregulated	2013	Induction of HIF-1α	IDH2	Microarray	23263745
miR-223	Upregulated	2013	Positive regulator of cell growth and invasion	PAX6	qRT-PCR	23970099
miR-143	Downregulated	2013	Inhibitor of glycolysis	HK2	qRT-PCR	23376635
miR-134	Downregulated	2013	Negative regulator of cell proliferation, invasiveness and migration. Positive regulator of apoptosis	NANOG	qRT-PCR	23467648

miR-203	Downregu-lated	2014	Inhibitor of cell prolifera-tion and in-vasion	PLD2	qRT-PCR	24270883
miR-138	Downregu-lated	2013	Negative regulator of cell cycle	EZH2, CDK6, E2F2, E2F3	qRT-PCR	23707559
miR-100	Downregu-lated	2013	Negative regulator of cell prolifera-tion	NCOR2	qRT-PCR	24244722
miR-219-5p	Downregu-lated	2013	Negative regulator of cell prolifera-tion, anchor-age inde-pendent growth and migration	EGFR	qRT-PCR	23690991
miR-29c	Downregu-lated	2013	Negative regulator of cell prolifera-tion, migra-tion and in-vasion. Posi-tive regula-tor of apop-tosis	CDK6	qRT-PCR	23744344
miR-495	Downregu-lated	2013	Negative regulator of cell prolifera-tion	CDK6	qRT-PCR	23594394
miR-153	Downregu-lated	2013	Negative regulator of cell growth and positive regulator of apoptosis	N/A	qRT-PCR	23397238
miR-328	Upregulated	2014	Positive reu-lator of cell invasion	SFRP1	qRT-PCR	24305703
miR-331-3p	Downregu-lated	2014	Inhibitor of cell migra-tion	NRP2	qRT-PCR	24142150
miR-106b-5p	Upregulated	2014	Positive reg-ulator of cell proliferation;	RBL1, RBL2, CASP8	Microarray; qRT-PCR	24166509

			negative regulator of apoptosis			
miR-106a	Downregulated	2013	Inhibitor of cell glucose uptake and proliferation	SLC2A3	qRT-PCR	24124917
miR-708	Downregulated	2013	Inhibitor of cell proliferation and invasion and positive regulator of apoptosis	Akt1, CCND1, MMP2, EZH2, Parp-1 and Bcl2	qRT-PCR	23754151
miR-24	Upregulated	2013	Positive regulation of cell proliferation and invasion	ST7L	qRT-PCR	23142218
miR-139	Downregulated	2013	Negative regulation of cell proliferation	Mcl-1	qRT-PCR	23551751
miR-34c-3p and miR-34c-5p	Downregulated	2013	Positive regulator of apoptosis; negative reguator of cell proliferation	Notch2	qRT-PCR	24179539
miR-503	Downregulated	2014	Inhibitor of cell proliferation, migration and invasion; positive regulator of apoptosis	IGF1R	qRT-PCR	24378652
miR-26a	Upregulated	2013	Positive regulation of cell proliferation	PTEN	qRT-PCR	24140063
miR-125a	Downregulated	2015	Inhibitor of cell proliferation; positive regulator of	TAZ	qRT-PCR	25542152

			GBM cell differentiation			
miR-148a	Upregulated	2014	Positive regulator of cell growth, survival, migration, and invasion	MIG6; BIM	qRT-PCR	24425048
miR-18a	Upregulated	2014	Positive regulator of cell proliferation, migration and invasion; negative regulator of apoptosis	NEO1	qRT-PCR	24657544
miR-377	Downregulated	2014	Negative regulator of cell proliferation and invasion	Sp1	Microarray; qRT-PCR	24951112
miR-127-3p	Downregulated	2014	Inhibitor of cell proliferation	SKI; RGMA ; ZWINT; SERPINB9; SFRP1	qRT-PCR	24517116
miR-671-5p	Upregulated	2016	Positive reulator of cell proliferation and migration	CDR1AS	qRT-PCR	26683098
miR-500a-5p	Upregulated	2018	Positive reulator of cell proliferation, migration and invasiveness	CHD5	qRT-PCR	30015879
miR-29a	Downregulated	2019	Inhibitor of cell stemness and cell growth; positive regulator of apoptosis	PDGFC; PDGFA	qRT-PCR	31482267
